# Sensitizing the Tumor Microenvironment to Immune Checkpoint Therapy

**DOI:** 10.3389/fimmu.2020.00223

**Published:** 2020-02-18

**Authors:** Rachael M. Zemek, Wee Loong Chin, Anna K. Nowak, Michael J. Millward, Richard A. Lake, W. Joost Lesterhuis

**Affiliations:** ^1^Telethon Kids Institute, University of Western Australia, West Perth, WA, Australia; ^2^National Centre for Asbestos Related Diseases, Nedlands, WA, Australia; ^3^Medical School, University of Western Australia, Crawley, WA, Australia; ^4^Department of Medical Oncology, Sir Charles Gairdner Hospital, Nedlands, WA, Australia; ^5^School of Biomedical Sciences, University of Western Australia, Crawley, WA, Australia

**Keywords:** cancer immunotherapy, immune checkpoints, sensitization, tumor microenvironment, PD-1, CTLA4, biomarkers, systems biology

## Abstract

Immune checkpoint blockade (ICB) has revolutionized cancer treatment, providing remarkable clinical responses in some patients. However, the majority of patients do not respond. It is therefore crucial both to identify predictive biomarkers of response and to increase the response rates to immune checkpoint therapy. In this review we explore the current literature about the predictive characteristics of the tumor microenvironment and discuss therapeutic approaches that aim to change this toward a milieu that is conducive to response. We propose a personalized biomarker-based adaptive approach to immunotherapy, whereby a sensitizing therapy is tailored to the patient's specific tumor microenvironment, followed by on-treatment verification of a change in the targeted biomarker, followed by immune checkpoint therapy. By incorporating detailed knowledge of the immunological tumor microenvironment, we may be able to sensitize currently non-responsive tumors to respond to immune checkpoint therapy.

## Introduction

Therapeutic approaches that inhibit negative regulatory immune checkpoints or stimulate activating immune checkpoints have shown great success in preclinical models and clinical trials (1, 2). In particular, antibodies that block cytotoxic T lymphocyte associated protein 4 (CTLA4), and programmed death 1 (PD-1) or its ligand PD-L1 have demonstrated unprecedented therapeutic efficacy in metastatic melanoma, non-small cell lung cancer, mismatch repair deficient cancers, and several other cancer types (3, 4). In these cancer types, significant response rates and survival benefits are seen, with a proportion of durable complete regressions, allowing the word “cure” to enter the oncologists' vocabulary (5). However, despite some positive outcomes, survival gains are modest in most cancers, and even in the most responsive cancers, many patients do not experience clinical benefit (6). This heterogeneity in response has led to a search for predictive biomarkers that could identify whether a patient will or will not respond to immune checkpoint blockade (ICB). In addition, although the targets for these antibodies are known, the down-stream biological consequences of therapeutic target engagement—both systemic and in the tumor microenvironment (TME)—are incompletely understood (6). Hence, although there is a clear need to improve the therapeutic efficacy of ICB, most clinical trials testing combination therapies are empirical, and often based on scant biological or preclinical data (7, 8).

In mouse cancer models using subcutaneous tumors derived from clonal cell lines we also observe a dichotomy in response to ICB; some mice experience complete response to treatment, while in others, their tumor continues to progress, despite having been treated under identical environmental and experimental conditions, including equal tumor burden and presumably identical (neo-)antigen expression (9–13). It is noteworthy that the probability of response in these mice can generally be increased by treating with ICB earlier, at a smaller tumor size (9). Together, these data suggest that each mouse has the capacity to respond, but that even potentially sensitive tumors may not respond if the pre-treatment conditions are not optimal. Furthermore, some murine tumor models never respond to ICB. Are these tumors intrinsically resistant, or do they lack functional TME attributes that could be therapeutically induced? Promisingly, some recent studies have been able to render otherwise resistant models sensitive to subsequent ICB (9, 14).

**Figure 1:**
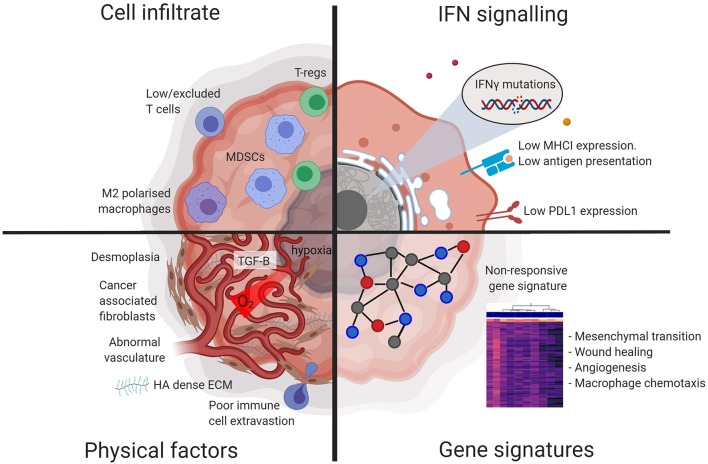
Diagram illustrating factors characteristic of a non-responsive tumor microenvironment. Tumors that are non-responsive to checkpoint blockade display resistance at the physical, cellular, protein, and gene expression level. Figures made in ^©^BioRender-biorender.com.

**Figure 2:**
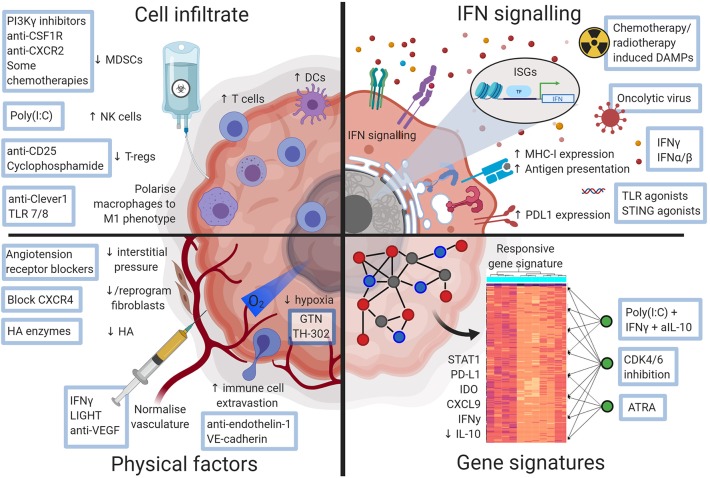
Diagram illustrating ways a checkpoint blockade favorable tumor microenvironment may be therapeutically attained. Approaches that could alter the non-responsive tumor microenvironment and sensitize tumors to checkpoint blockade. Figures made in ^©^BioRender-biorender.com.

Despite known associations between pre-treatment TME characteristics and response [see references (15) and (16) for comprehensive reviews of predictive biomarkers], strategies to induce a responsive phenotype and thus sensitize cancers to ICB are only beginning to be developed. Systemic immunity and the local immune response at the effector site are obviously linked, and indeed several studies, both in animal models and patients, have shown that a degree of systemic immunity is required for tumors to respond to ICB (17, 18). Here, we focus on factors within the TME: we summarize and contextualize recent studies characterizing the features of an ICB responsive, contrasting with a non-responsive, TME (Figure 1), and discuss selected therapeutic interventions designed to modulate that environment toward a responsive phenotype (Figure 2).

## Turning “Cold” Tumors “Hot”—Cells in the Responsive TME

Recently, the concept of hot vs. cold tumors has become widely accepted in immuno-oncology, where “hot” denotes tumors that contain more than a defined threshold of inflammatory cells, while “cold” tumors do not. Other terms characterizing response-associated cellular TMEs include describing cold tumors as either “immune desert” (absence of immune cells) or “infiltrated-excluded” (tumors with only peripheral invasion of immune cells); and classifying “hot” tumors into those with tertiary lymphoid structures, or “infiltration-inflamed” tumors (inflammatory myeloid cells and activated CD8^+^ T cells) (19, 20). Alternatively, more comprehensive characterizations incorporate several different aspects of T cell immunity resulting in more subcategories and allowing a scoring metric to be applied (21, 22). A potential caveat of these approaches is that innate immune cells are underrepresented. Accumulating evidence indicates that innate immune cells play a role in the responsive TME and could be exploited therapeutically to improve outcomes. Relevant cells include macrophages (23–25), dendritic cells (DCs) (26) and natural killer (NK) cells (9, 26). We will briefly discuss several cell types in the TME associated with response.

### T Cells

Experiments in murine cancer models have consistently demonstrated the importance of T cells for the efficacy of ICB (27–29). In humans, secondary resistance to ICB has also been associated with T cell deficits, including mutations in cancer cells associated with decreased sensitivity to T cell-mediated killing, or reduced antigen presentation to T cells (30, 31). Baseline numbers of tumor-infiltrating lymphocytes (TILs) have been found to correlate with response to anti-PD-1 alone, but not with combination ICB therapy (32–34). CD8^+^ T cells have been shown to be the anti-tumor effector cells, and sensitivity to ICB was enhanced in tumors enriched for CD8^+^ T cells reactive to clonal neoantigens (35). The differential role of CD8^+^ vs. CD4^+^ T cells is less clear, with conflicting outcomes in different tumor models (28, 29). The interpretation of these results is difficult because CD4 depletion in mice not only depletes T effector cells, but also regulatory T cells (T-regs) (36). It should be noted that anti-mouse PD-1 antibodies used in murine models are often raised in rats, and repeated dosing will result in anti-antibody formation. In addition, there are important differences in IgG isotype and their affinity for Fc receptors (37, 38), which may partly explain the difference in efficacy between murine and human studies.

Pre-treatment with selected chemotherapeutics has been shown to enhance T cell responses in the experimental setting (39). For example, oxaliplatin plus cyclophosphamide treatment of lung adenocarcinomas increased the ratio of CD8+ T cells vs. T-regs, increased the presence of tumor-specific CD8+ T cells, and resulted in enhanced expression of PD-1 and PD-L1 with subsequent improved responsiveness to ICB (40). Similarly, gemcitabine only synergized with CD40 directed immunotherapy when mice were pre-treated with gemcitabine, but it was not effective when given concurrently or after immunotherapy (41).

T-regs express high levels of CTLA4, and antibodies blocking CTLA4 can deplete T-regs in the TME in murine models, dependent on Fc subclass and host Fc receptor (42–44). T-reg depletion has also been hypothesized as a key mechanism of action for anti-CTLA4 treatment in humans (45, 46), however clinical data does not support this (47), which may be due to the different isotype and Fc portion of the human antibody. Indeed, there is an association between pre-treatment Foxp3+ T-reg infiltration, followed by a subsequent increase in TILs 3 weeks into treatment in melanoma biopsies which was associated with response to the CTLA4-targeting antibody ipilimumab (48). Whether this is a causal relationship, or a bystander effect of enhanced inflammation remains to be established, as infiltration of the TME with T-regs usually coexists with an inflammatory response that also includes CD8^+^ T cells, macrophages and granulocytes (49, 50). Conversely, T-regs and T effector cells express similar levels of PD-1 (51), and anti-PD-1 antibodies do not deplete cells expressing the target, but the Fc portion can modulate myeloid cell activity (37). Depletion of T-regs, nevertheless, may enhance the efficacy of ICB. Depletion of T-reg using an Fc-optimized anti-CD25 antibody prior to anti-PD-1 treatment resulted in a greater response rate in a murine model, indicating that depleting T-regs prior to ICB may be viable sensitization strategy (52). An intriguing approach turned T-regs into interferon (IFN)γ-producing T effector cells by targeting the CARMA1-BCL10-MALT1 signalosome complex. This resulted in an inflammatory TME with increased expression of MHC class II by macrophages and MHC class I and PD-L1 by tumor cells, facilitating increased T cell mediated tumor lysis (53). Another strategy to target Treg is pharmacological inhibition using the allosteric MALT1 inhibitor mepazine (previously used in psychiatric diseases). Combination therapy with mepazine and PD-1 blockade resulted in an additive anti-tumor effect (53). However, as yet there are no clinically available therapeutic agents which deplete human T-reg.

### Macrophages

Macrophages may also play a role in response to ICB, although both tumor-promoting and tumoricidal effects have been noted. Macrophages are enriched in anti-PD-1 resistant human non-small cell lung cancer (NSCLC) (24). Conversely, macrophages have also been noted in the regression bed of neoadjuvant treated NSCLC patients with complete response (25). These seemingly opposing effects may be partially explained by the heterogeneity of macrophages, which can change polarization from a pro-tumorigenic M2 phenotype to a more tumoricidal M1 phenotype, a phenomenon which could be exploited therapeutically. Viitala et al. identified common lymphatic endothelial and vascular endothelial receptor-1 (Clever-1) as a driver of M2 polarization. Treatment of Lewis lung cancer-bearing mice with an antibody targeting Clever-1 concomitant with PD-1 blockade provided a modest additive anti-tumor effect (54). Similarly, Rodell et al. used Toll-like receptor 7/8 agonist-loaded nanoparticles to polarize macrophages toward an M1 phenotype, resulting in single-agent anti-tumor efficacy, and additive effects in combination with PD-1 blockade in mice bearing MC38 or B16 tumors (55). Notably, these studies used concomitant treatments and did not explore sensitization strategies using sequential scheduling of treatment to alter the TME prior to ICB.

### Myeloid Derived Suppressor Cells

Myeloid-derived suppressor cells (MDSCs) have been linked to a reduced efficacy of several immune-based cancer therapies (56–60), and therefore can also be targeted to enhance ICB efficacy. In murine cancer models, the gamma isoform of phosphoinositide 3-kinase (PI3Kγ), which is highly expressed in myeloid cells, can be targeted with a selective inhibitor that reprograms MDSCs and improves responses to antibodies targeting CTLA4, PD-1, or both (61). Selective PI3Kγ inhibitors are currently being evaluated in clinical trials (62). Similarly, blocking CSF1R prevents MDSCs from exerting immunosuppressive effects, enhances anti-tumor T-cell responses, and improves response to checkpoint blockade in several murine models to ICB (63–65). Other approaches include blocking the chemokine receptor CXCR2 to prevent MDSC recruitment into the tumor, which sensitizes a mouse model of rhabdomyosarcoma to anti-PD-1 (66), and using the repurposed drug ibrutinib which inhibits MDSCs and sensitizes murine breast cancer models to anti-PD-L1 (67, 68).

### Natural Killer Cells

There has been increasing interest in the role of NK cells in anti-tumor immunity, and the potential to modulate their function therapeutically. In human nasopharyngeal cancer, the presence of functionally exhausted NK cells predicted worse outcomes, and reversing NK cell exhaustion *in vitro* restored anti-tumor effects (69). Furthermore, a high number of intra-tumoral NK cells in patient melanoma samples at various stages of treatment predicted responsiveness to anti-PD-1. NK cell associated genes were correlated with expression of *Flt3lg* in the Cancer Genome Atlas melanoma dataset, suggesting a DC stimulatory role for NK cells (26). Although NK cells may not be required for the direct anti-tumor effects driven by ICB, they appear to play a role in supporting an immune-favorable TME. NK cells are required for the accumulation of conventional type I dendritic cells (cDC1) into tumors in mouse models, which are crucial for T cell anti-tumor immunity (70). In addition, we recently identified higher numbers of activated NK cells in the pre-treatment TME of responsive tumors in mouse models treated with anti-CTLA4/anti-PD-L1 compared with those that did not respond. This observation was validated in data from patients treated with anti-PD-L1 (9, 71). Depletion of NK cells prior to ICB abrogated the response, confirming a role of NK cells in the priming of the TME, potentially through local IFNγ production (9). Pre-treatment with poly(I:C), IFNγ and anti-IL-10 increased NK infiltration and sensitized four different tumor models to subsequent ICB. This sensitizing effect was similarly abrogated when NK cells were depleted, despite intratumoral IFNγ administration, suggesting that the effect of NK cells was not restricted to local IFNγ production (9).

In conclusion, the concept of hot vs. cold continues to be useful to explain some differential sensitivity to ICB. However, as more granular information emerges about the functionality of infiltrating cells in the TME and how this is associated with response, this binary description may miss some nuances. For example, in a homogenous background, using tumors derived from clonal cancer cell lines in inbred mice, we found that the composition of cellular infiltrates between responsive and non-responsive tumors was largely identical for CD8^+^ T cells, macrophages, DCs and granulocytes (9). However, cells in the TME of responding mice had a more activated phenotype as measured by MHCI, PD-L1 and activated regulatory networks, compared to non-responding mice (discussed in more detail below) (9). Specific characteristics of infiltrating cells, such as cellular phenotype and activation state, may define the sensitive or resistant nature of the TME more than cell lineage or origin. The examples above show that therapeutic approaches that change these phenotypic and functional characteristics are able to sensitize tumors to ICB (Figure 2).

## Sensitizing the TME Through Enhanced IFN Signaling

Interferons have been associated with both responsiveness and resistance to immunotherapy (72). Expression of IFNγ and IFNγ-inducible genes, such as IDO and CXCL9, are positive biomarkers of response to ICB (73). Upregulation of IFNγ can promote T cell responses, as well as upregulate MHC class I molecules on tumor cells, increasing their sensitivity to cytotoxic T cells (40, 74, 75). At the same time, IFNγ upregulates PD-L1 on cancer cells, leading to the possibility of T cell exhaustion. Targeted activation of the type I IFN system (IFN α and β) renders resistant immune-cell poor tumors sensitive to ICB (76). Some chemotherapies also upregulate type I IFNs, attracting T cells to the TME (77). Combination anti-CTLA4 and anti-PD-1 work synergistically by increasing IFNγ signaling, which in turn increases IL-7 signaling, resulting in superior tumor eradication (78). Inducing IFN signaling may therefore be exploited to increase response to ICB. However, prolonged activation of type I IFN can induce resistance to ICB through stimulation of nitric oxide synthase 2 (NOS2), resulting in increased infiltration of Treg and myeloid cells (79). The finding that IFN-pathways can drive resistance has been reported by Benci et al. (80). Using a B16 cell line that progressed after ICB, they found that resistance was due to enhanced IFNγ/PD-L1 signaling in the tumor cells. Specific blockade of IFNγ signaling in tumor cells resulted in increased sensitivity to PD-1 blockade in an NK/ILC1-dependent manner. This is mirrored by the observation that genetic deletion of the type I IFN pathway in cancer cells increases responses to ICB (81). Interestingly, by inhibiting tumor derived IFNγ and decreasing the immune stimulated genes in tumor cells, IFNγ production by T cells was increased, promoting tumor cell killing (82). In addition, prolonged IFN signaling can drive clonal selection leading to recurrence after an initial response to ICB. In the clinical setting, defects in the pathways involved in interferon-receptor signaling favored outgrowth and sequential progression in melanoma patients treated with anti-PD-1(83, 84). Together, these data indicate that stimulation of the IFN system is a balance between priming the TME to subsequent ICB or rendering it resistant. The spatial and temporal characteristics of the IFN response likely play a role in this outcome.

### Cytosolic Sensor Activation

Interferons can be upregulated via several mechanisms. Activation of cytosolic sensors by pathogen-associated molecular patterns (PAMPs) or damage-associated molecular patterns (DAMPs) results in a robust IFN response. These DAMPs can be released as a consequence of treatment with some chemotherapeutics or radiotherapy, resulting in increased production of type I and II IFNs in the TME (85, 86). Interestingly, a *post-hoc* analysis of the KEYNOTE-001 trial of NSCLC patients treated with anti-PD-1 suggested that patients who had received radiation prior to anti-PD-1 had significantly longer survival than those who did not (87).

The stimulator of interferon genes (STING) sensor can be activated by free DNA, which is released after radiotherapy (88), or by direct injection of cyclic dinucleotides (89), resulting in strong immune activation which can overcome tumor immune suppression (88, 90). Similarly, Toll-like receptors (TLRs) such as TLR3 and TLR9 can be activated by poly(I:C) and CpG, respectively, which can result in a potent inflammatory response when injected into tumors, and can induce long-lasting CD8^+^ T cell-dependent anti-tumor responses (76, 91). Activation of these cytosolic sensors results in type I IFN production, which as described above can control anti-tumor immunity (76, 92). There is experimental evidence that induction of IFN in the TME via activation of Toll-like receptors (TLR) increases the effectiveness of ICB. Peri-tumoral injection of TLR9 ligand CpG increased sensitivity to anti-PD-1 in murine models of bladder cancer (93). Similarly, several studies identified that TLR3 ligand poly(I:C) improved response rates to ICB in murine models of melanoma, lung, and colon cancer (76, 94). Importantly, these beneficial effects of poly(I:C) are schedule-dependent. A short course of treatment with a Poly(I:C)-based therapeutic combination prior to ICB sensitized several tumor models to anti-CTLA4/anti-PD-L1. However, treatment with ICB first, followed by poly(I:C) was not additive over ICB alone, emphasizing the importance of temporal aspects of an effective anti-tumor response, and by extension, of scheduling drugs in combination immunotherapy (9). There are ongoing clinical trials combining polyICLC [a stable derivative of poly(I:C)] with ICB (clinicaltrial.gov numbers: NCT02834052, NCT03721679, NCT02643303). Treatment with immunostimulatory molecules prior to ICB is a rational strategy to impose a sensitive phenotype onto tumors, however, they must be applied directly into the tumor, which may not always be feasible. A novel small molecule STING agonist has recently been developed for systemic use, which may overcome this limitation (95).

### Oncolytic Virotherapy

Oncolytic virotherapy is another strategy which has been used to induce IFN. Combining oncolytic virotherapy and CTLA4 blockade resulted in rejection of distant (non-virally injected) tumors in the poorly immunogenic B16 melanoma model, which was dependent on NK cells and type I IFN (96). Oncolytic virotherapy was also able to sensitize a model of triple-negative breast cancer to ICB (97). In the clinical setting, intravenous oncolytic *Orthoreovirus* increased T cell infiltration in primary and metastatic brain cancer and up-regulated IFN-regulated gene expression and PD-L1 expression, creating a favorable TME for subsequent ICB therapy (98). Another clinical trial using an attenuated herpes simplex virus type 1, followed by anti-PD-1 therapy for melanoma patients resulted in a 33% complete response rate, with increased CD8^+^ T cells, and increased PD-L1 protein and IFNγ gene expression in responders. Baseline CD8^+^ T cell infiltration or a baseline IFNγ signature was not associated with response (99). Oncolytic viral therapy is therefore a potential future option to skew the TME toward an ICB responsive phenotype, provided that at least some of the tumor is accessible for local injection.

## Altering Physical Factors of Tumors to Improve Response

Recent research has highlighted that, besides biological, and chemical cues from the microenvironment, physical cues can also greatly alter cellular behavior of cancer cells. Abnormal vasculature and barriers to perfusion, such as high interstitial pressure within tumors can antagonize the effectiveness of ICB by promoting TME-mediated immune suppression. The impaired perfusion capacity of tumor blood vessels helps to create an immune cell unfavorable TME (100, 101). However, these physical characteristics can vary greatly between tumor types. For example, biomarkers used for typical ICB-responsive cancer-types, such as mutational burden, level of tumor-infiltrating T lymphocytes or expression of immune-checkpoints are not predictive for glioblastoma, partly due to the different physical environment (102).

### Normalizing Tumor Vasculature and Immune Cell Infiltration

Responsive tumors are characterized as having greater immune cell infiltrate, and the baseline infiltration level of T cells has important predictive and prognostic implications for ICB (103, 104). Although activated T cells are observed in the periphery of non-responsive tumors after ICB, they often fail to infiltrate the tumor itself (105). Tumors exploit many mechanisms to limit immune cell infiltration. Proangiogenic growth factors downregulate adhesion molecule expression, limiting extravasation of immune cells across the tumor endothelium (101). The tumor endothelium can be manipulated through selective blockade of angiogenic factors including VEGF and endothelin-1 (105, 106), or by increasing VE-cadherin expression (107), resulting in increased T-cell infiltration. Combination blockade of VEGFA and angiopoietin-2 normalized tumor blood vessels and increased lymphocyte infiltration, improving outcomes when combined with anti-PD-1 in B16-OVA and MC38 mouse models (108). A small clinical study in 10 patients with metastatic renal cell carcinoma found that pre-treatment with the VEGF blocking drug bevacizumab followed by a combination of bevacizumab and PD-L1 resulted in a 40% response rate, which was high compared to historical controls for either agent alone (109). A unique approach using vascular targeting peptides allowed targeted delivery of the pro-inflammatory cytokine LIGHT to tumor vessels in murine models (110). This approach normalized tumor vasculature and increased intratumoral effector T cells when combined with ICB, resulting in responses in otherwise immunotherapy-resistant tumors.

Abnormal tumor vasculature promotes resistance to ICB via various effects. Abnormal vasculature limits access of ICB antibodies into the TME and reduces oxygen availability, leading to a hypoxic TME. Hypoxia in turn, upregulates several immune checkpoints in the TME, including PD-L1, CD47, VISTA and 4-1BB (CD137), impairing anti-tumor responses. Furthermore, hypoxia facilitates recruitment of MDSCs and enhances their immunosuppressive function (111). The immunosuppressor TGF-β1 is promoted by overexpression of HIF-1α in tumor hypoxia, which fosters exclusion of T lymphocytes (71, 112). Targeting hypoxia-induced immunosuppression improves outcomes from ICB, but there are few studies addressing whether targeting hypoxia directly sensitizes to ICB. In one study, *in vitro* pre-treatment of B16-OVA tumor cells with GTN, an agonist of nitric oxide, inhibited hypoxia-induced resistance to CTL-mediated lysis (113). In another, combining the hypoxia reducing prodrug (TH-302) with checkpoint blockade in a mouse prostate cancer model significantly reduced the number of tumor infiltrating MDSCs and cured around 80% of mice (114).

### Circumventing High Interstitial Pressures in Tumors

Another feature of the TME is “solid stress,” defined as a growth-induced increase of physical pressure, commonly exacerbated by overproduction of hyaluronic acid (HA). This leads to high interstitial pressure and vessel collapse which may result in the exclusion of immune cells (115). Treatment with pegvorhyaluronidase alfa, which enzymatically degrades HA, has been tested to ameliorate solid stress, increasing both infiltration of immune cells and intratumoral uptake of anti-PD-L1 antibodies. Pre-treatment with pegvorhyaluronidase alfa 24 h prior to anti-PD-L1 resulted in significant growth inhibition in an anti-PD-L1-resistant breast cancer model which was genetically engineered to express hyaluronan synthase 3 (116). Desmoplasia, the growth of fibrous or connective tissue produced by cancer-associated fibroblasts, can also contribute to solid stress. A dense fibrotic stroma is associated with poor prognosis and is a common feature of immunotherapy resistant tumors such as pancreatic and breast cancer. One approach to decrease tumor interstitial pressure is to reprogram cancer associated fibroblasts to a quiescent state using angiotensin receptor blockers. Treatment with tumor-targeted angiotensin receptor blockers increased tumor perfusion, reduced immunosuppression, and enhanced the efficacy of anti-PD-1 treatment (117). Fibroblast function is also modulated by hypoxia, which induces signaling by CXCL12 via CXCR4, which promotes CAF recruitment, activation, and matrix production. Inhibition of CXCR4 signaling alleviated solid stress and increased the response to anti-CTLA4/anti PD-1 ICB in three metastatic breast cancer models (118).

## Altering the Microbiome to Augment Immune Cells Within the TME

The microbiome has been shown to directly impact the TME, as antibiotic-treated or germ-free mice have defective tumor-infiltrating myeloid-derived cells with lower cytokine production and tumor necrosis after CpG-oligonucleotide treatment compared to controls (119). Conversely, commercialization with the bacterial species *Bifidobacterium* improved dendritic cell function and subsequent tumor-killing capabilities of cytotoxic T cells, resulting in reduced growth of subcutaneous melanoma xenograft models in mice. Additionally, *Bifidobacterium* administration in combination with anti-PD-L1 nearly abolished tumor growth (120). Other bacterial species such as *Bacteroides thetaiotamicron* and non-toxigenic *B. fragilis* have shown to improve antitumor cytotoxic T-cell immunity improving the efficacy of anti-CTLA-4 in multiple cancer mouse models (121). In melanoma patients treated with anti-PD-1, a positive correlation was found between the number of tumor infiltrating CD8^+^ T cells and the abundance of *Faecalibacterium* in responders (122). Several studies have found a link between the fecal microbiome and response to ICB (120–125). Fecal transplants from responding patients into mice has shown to improve tumor control, augment T cell responses, and increase the efficacy of anti-PD-L1 therapy (124) or PD-1 therapy (125), showing promise as a potential sensitizing strategy.

## Modulating Gene Signatures Toward a Permissive TME

In the context of immune checkpoint blockade, important biological changes in the tumor microenvironment are reflected in changes in gene expression. Gene expression data from pre-treatment melanoma samples revealed immune-related signatures that were highly expressed in patients whose tumors responded to anti-CTLA4 or anti-PD-1 therapy, compared to non-responders (73, 126). In another study using bulk RNAseq data, non-responders to anti-PD-1 had higher pre-treatment expression of genes involved in mesenchymal transition, immunosuppression (including genes associated with wound healing and angiogenesis), and monocyte and macrophage chemotaxis (127). More recently, time-dependent transcriptomic changes have been associated with response to checkpoint blockade. RNA sequencing before and during anti-PD-1 therapy showed that tumor samples from responsive patients displayed upregulation of immune checkpoint genes and activation of response-specific transcriptional networks (128). Taken together, these results highlight the importance of transcriptomic changes in the tumor microenvironment. Moreover, it raises the possibility that manipulating gene expression patterns in the tumor microenvironment will impact treatment with ICB.

As proof of concept of this approach, gene expression changes in non-responsive tumors to ICB were used to inform computational drug discovery. Using single cell data of 33 melanoma patients, Jerby-Arnon et al. identified a transcriptional program expressed in malignant melanoma cells indicating a poor response to checkpoint blockade (129). They linked this poorer response to a less permissive TME, as evidenced by intratumoral niches of T-cell depletion. In a drug screen on 131 cell lines, they found their transcriptional signature to be antagonized by CDK4/6-inhibitors such as abemaciclib and palbociclib. Combining CDK4/6-inhibitors with checkpoint blockade inhibited tumor outgrowth in mouse models of melanoma, validating the approach of using gene signatures and bioinformatics interrogation to identify effective combination therapies (129).

In our own work, we used a systems biology approach to contrast responsive and non-responsive tumors prior to therapy in two murine models, to identify upstream regulators of the gene signature response signature to ICB (6, 9, 10). We found that responsive tumors were characterized by an inflammatory gene expression signature consistent with upregulation of STAT1 and TLR3 signaling, and down-regulation of IL-10 signaling. Therapeutically targeting these drivers using poly(I:C), IFNγ and an anti-IL-10 monoclonal antibody sensitized the TME and significantly increased response to ICB in multiple murine models. The triple combination was superior to any of the single drugs, validating the approach to identify complex therapeutic combinations for tumor sensitization (9). Similarly, by interrogating drug repurposing databases for the response-associated gene expression profile of anti-CTLA4 treated mice, we identified all-trans retinoic acid as a potential drug candidate to improve outcomes to ICB therapy. Treatment with all-trans retinoic acid indeed significantly increased the response rate when combined with anti-CTLA4 over either treatment alone (10). Bioinformatics approaches will facilitate rapid identification of new ways to sensitize the TME.

## Discussion

### Going Forward: Personalized Biomarker-Based Adaptive Therapy

**Figure 3:**
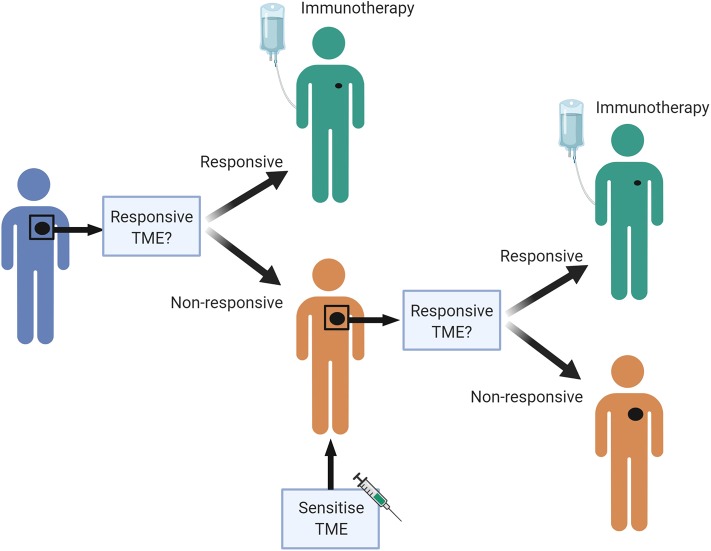
A personalized pre-treatment strategy to optimize outcomes to immune checkpoint blockade. We propose a pre-treatment approach to treating cancer patients, where tumors displaying features of a non-responsive TME can be first sensitized to attain a favorable TME, to improve chances of response to immune checkpoint blockade. Figures made in ^©^BioRender-biorender.com. Adapted from a figure we published recently in Science Translational Medicine (7).

There are likely to be many different ways to increase the chance that a patient will respond to ICB, and these are likely to differ between individuals. A personalized pathway to improve treatment effectiveness may be possible in the future (Figure 3). We propose that a pre-treatment biopsy will determine the baseline TME. If the patient's tumor has many characteristics associated with a responsive TME, they can start treatment with ICB. If not, a sensitizing therapy could be considered first, based on the genetic and immunological profile of the tumor. If, for example, the biopsy demonstrates many macrophages, but little IFN/STAT1 signaling, therapy aimed to polarize macrophages could be considered; if there are many T-regs in the biopsy, therapy aimed at reducing those could be considered. An on-treatment biopsy shortly after initiation of the sensitization strategy can verify whether the biological endpoint has been achieved. If so, the patient can progress to treatment with ICB. If not, another sensitization strategy could be considered, or another therapy altogether. Although this proposal is attractive, and would prevent patients from undergoing potentially futile therapy with attendant physical and financial toxicity, none of the biomarkers discussed are currently robust enough to justify withholding ICB in patients with an appropriate indication, and none of the sensitization strategies have been clinically validated in prospective trials.

How can we progress toward personalized immunotherapy and sensitization? Firstly, preclinical studies must include more robust study of scheduling effects. As the anti-tumor immune response is a highly orchestrated program involving many cells from both innate and adaptive immunity, changing over time, it is likely that temporal aspects are important for optimal immunological control, similar to the antiviral response (6, 130). However, as discussed above, most murine cancer studies do not rigorously study scheduling when investigating combination treatments. Secondly, human *window of opportunity* trials could help screen for drugs that induce a response-associated TME, by making use of the short period between cancer diagnostic biopsy and primary surgery (131). Sequential biopsies in clinical trials can also provide more insight into how drugs change the TME. Lastly, bioinformatics approaches will also need to continue to develop, to improve our understanding of a responsive TME (132).

The future goal of this personalized biomarker-based adaptive treatment approach is to give each patient the best chance of a successful response to immunotherapy.

## Author Contributions

RZ and WL conceptualized and wrote the review. WC contributed to sections of the review. RL, AN, and MM provided critical review and editing of the manuscript. RZ designed and generated the figures. All authors contributed to manuscript revision, read, and approved the submitted version.

### Conflict of Interest

Patent application pertaining to aspects of discussed work: RZ, RL, and WL (PCT/AU2019/050259, “Method for immunotherapy drug treatment”). AN, MM, RL, WL research funding and consultancy for Douglas Pharmaceuticals. WL research funding from AstraZeneca. AN consultant or advisory boards for Boehringer Ingelheim, Bayer Pharmaceuticals, Roche Pharmaceutics, Merck Sharp Dohme, Pharmabcine, Atara biotherapeutics and Trizell; research funding from AstraZeneca. MM advisory boards for Merck Sharp & Dohme, Bristol-Myers Squibb, Roche, Astra-Zeneca. The remaining author declares that the research was conducted in the absence of any commercial or financial relationships that could be construed as a potential conflict of interest.
